# Generation of Human Neutrophils from Induced Pluripotent Stem Cells in Chemically Defined Conditions Using *ETV2* Modified mRNA

**DOI:** 10.1016/j.xpro.2020.100075

**Published:** 2020-07-27

**Authors:** Aditi Majumder, Kran Suknuntha, David Bennin, Lucas Klemm, Vera S. Brok-Volchanskaya, Anna Huttenlocher, Igor Slukvin

**Affiliations:** 1 Wisconsin National Primate Research Center, University of Wisconsin, Madison, WI 53715, USA; 2 Department of Pathology and Laboratory Medicine, University of Wisconsin, Madison, WI 53792, USA; 3 Chakri Naruebodindra Medical Institute, Faculty of Medicine, Ramathibodi Hospital, Mahidol University, Samut Prakan 10540, Thailand; 4 Department of Pediatrics and Medical Microbiology and Immunology, University of Wisconsin-Madison, Madison, WI 53706, USA; 5 Department of Cell and Regenerative Biology, University of Wisconsin School of Medicine and Public Health, Madison, WI 53707-7365, USA

## Abstract

This protocol describes a rapid and efficient feeder-, serum-, and xeno-free method for neutrophil generation from hiPSCs using *ETV2* modified mRNA (mmRNA), which directs hematoendothelial programming of hiPSCs. Hematoendothelial progenitors were cultured with GM-CSF, FGF-2, and UM171 to expand myelomonocytic progenitors, followed by treatment with G-CSF and retinoic acid agonist Am580 to induce neutrophil maturation. This protocol is suitable for generating functional neutrophils from iPSCs to interrogate the role of genes in a neutrophil development and function.

For complete details on the use and execution of this protocol, please refer to Brok-Volchanskaya et al. (2019).

## Highlights

• A rapid and robust protocol for neutrophil production
• Induction of hematoendothelial program with ETV2 mmRNA
• Defined conditions for neutrophil differentiation without feeder cells or serum

## Graphical Abstract

**Figure:**
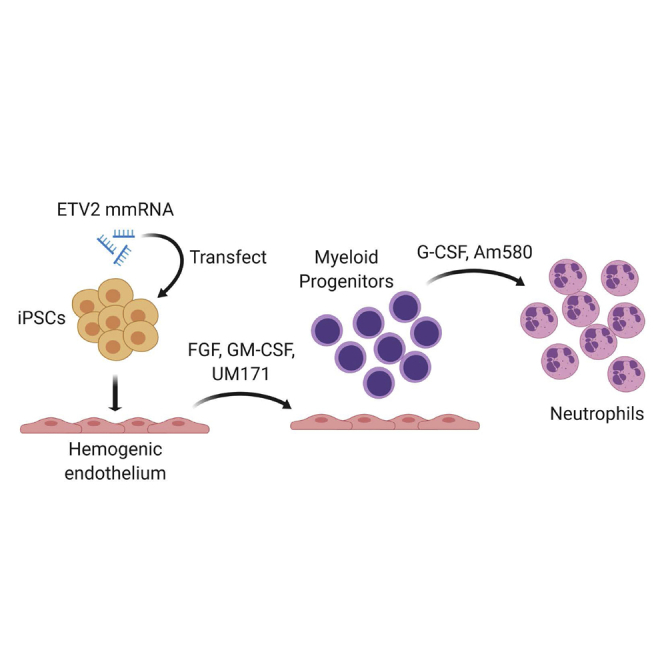

## Before You Begin

### Establish a Construct for ETV2 mmRNA Production

**Timing: ∼9 days**

**Figure 1:**
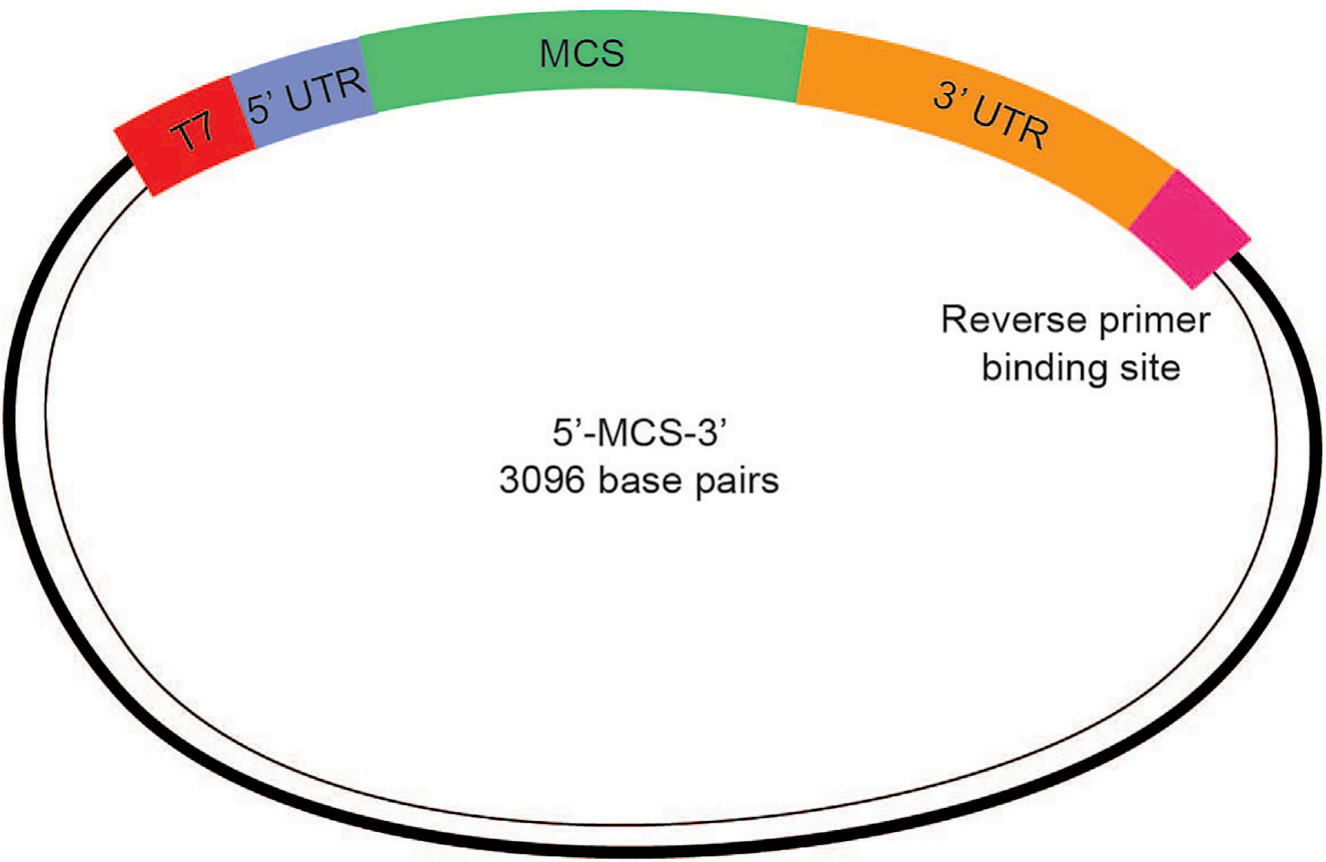
Schematic Representation of the 5′-MCS-3′β-Globin Construct for *ETV2* mmRNA Synthesis MCS is multiple cloning sites.
d. Using a high-fidelity DNA polymerase, *e.g.,* Q5 or Phusion, perform PCR with a reverse primer containing 180T nucleotides and forward primer ATCGGTGCGGGCCTCTTCGCTA, including T7 promoter, for generation of IVT templates with 180-A tract.
e. PCR for *ETV2* amplification:

1. Modification of vector for *in vitro* transcription (IVT) from pGEM-T Easy
a. Synthesize 5′ UTR of β-globin, multiple cloning sites (MCS), and 3′ UTR cassette.
b. Clone 5′ UTR of β-globin, multiple cloning sites (MCS), and 3′UTR cassette into pGEM-T Easy to generate 5′-MCS-3′β-globin construct (Suknuntha et al., 2018).
c. Perform cloning of human *ETV2* transcript variant 1 (NM_014209.3) into 5′-MCS-3′β-globin construct (Figure 1) to generate IVT template for *ETV2.*

**Table:** 

PCR Cycling Conditions			
Step	Temperature	Time	Cycles
Initial Denaturation	98°C	30 s	1
Denaturation	98°C	15 s	30
Annealing	54°C	15 s	
Extension	72°C	1 min	
Final extension	72°C	10 min	1
Hold	4°C	Forever

f. Run PCR product on agarose gel.
g. Extract the product using QIAEX II gel extraction kit (https://www.qiagen.com/us/resources/resourcedetail?id=13d33145-9f64-426a-a43b-394211d8cf2b&lang=en).
2. Synthesis of *ETV2* mmRNA
a) Use MEGAscript T7 kit for synthesis of ETV2 mmRNA with ribonucleotide cocktail containing 3′-O-Me-m7G(5′)ppp(5′)G ARCA cap analog, adenosine triphosphate, pseudouridine triphosphate and guanosine triphosphate, according to the manufacturer’s instructions.
b) Incubate reaction for 2 h at 37°C followed by DNase and recombinant shrimp alkaline phosphate (rSAP) treatment.
c) Prepare a working concentration of 100 ng/μL RNA in RNase-free water, aliquot into tubes and store at −80°C.

**CRITICAL:** RNase-free reagents and RNase inhibitor should be used to stabilize synthesized RNA and prevent degradation. RNase contamination is the most common cause of poor mmRNA recovery. Usually 30–40 μg of mmRNA can be synthesized using 500–1,000 ng of template per a 20 μL reaction. Control template (provided in the kit) can be performed in parallel for troubleshooting.

d) *ETV2* mmRNA transfection master mix:
i) Transfer aliquot of *ETV2* mmRNA from −80°C and place on ice (Figure 4B).
ii) Place TransIT®-mRNA Kit on ice from 4°C.
iii) For each well of a 6-well plate, combine the following reagents in a sterile 1.5 mL microcentrifuge tube at room temperature (Figure 4B):
100 μL TeSR™-E8™ complete media
0.4 μL TransIT reagent (Provided in TransIT®-mRNA Kit)
0.4 μL mRNA boost reagent (Provided in TransIT®-mRNA Kit)
200 ng *ETV2* mmRNA

Mix by gently pipetting and keeping at room temperature for 3 min before adding into respective well (Figure 4C).

### Production of Matrigel-Coated Plates

**Timing: ∼60 min**

3. Use Matrigel for culturing of hiPSCs. Matrigel™, Growth Factor Reduced is frozen at −20°C to −80°C. Thaw overnight on ice at 4°C.

***Note:*** Matrigel™ is liquid at 4°C and gels rapidly at room temperature.

4. Open the metal seal on the Matrigel™ bottle and carefully remove the rubber stopper. Place bottle on ice and aliquot 0.5 mg into prechilled microcentrifuge tubes and store at −80°C for up to 12 months.
5. In a sterile 15 mL conical tube add cold 11 mL of 1× DPBS.
6. Remove one 0.5 mg Matrigel™ aliquot from the freezer. Use a prechilled 1,000 μL pipet tip to add 1 mL of cold PBS to the Matrigel™ aliquot.
7. Gently pipet up and down to thaw and dissolve the Matrigel™. Immediately transfer it to the 15 mL conical tube with 11 mL of DPBS and pipet to mix.
8. Immediately plate 2 mL into each well of a 6-well plate. Store the plates at 4°C for up to 7 days.
9. Prior to use, incubate a Matrigel™ coated plate for at least 1–2 h at 37°C.
10. Aspirate the Matrigel™ and wash once with 1 mL of TeSR™-E8™ complete media.
11. Add 2 mL of TeSR™-E8™ complete media.

### Production of Collagen IV-Coated Plate

**Timing: ∼60 min**

12. To make 2 mg/mL of Collagen IV, dissolve 5 mg Collagen IV powder in 6.25 μL of acetic acid and 2.5 mL of sterile water and store overnight at 4°C. Aliquot 60 μL (2.4 μg/mL) into microcentrifuge tubes and store at −80°C for up to 12 months.
13. Remove one 60 μL aliquot from storage. Use a micropipette with a 1,000 μL tip to add 1 mL of ddH_2_O into the 60 μL aliquot of collagen IV and take the full mixture into 49 mL of ddH_2_O. It is enough for 4×6-well plates.
14. Immediately plate 2 mL in each well of a 6-well plate. Store the plates at 4°C for up to 7 days.
15. Prior to use, incubate for at least 1 h at 37°C.
16. Aspirate the collagen solution and wash once with 1 mL TeSR^TM-^E8™ complete media.
17. Add 2 mL TeSR™-E8™ complete media to each well.

## Key Resources Table

**Table:** 

REAGENT or RESOURCE	SOURCE	IDENTIFIER
**Antibodies**		

Mouse anti-human CD34-PE	BD Pharmingen™	Cat#555822
Mouse anti-human CD43-BV421	BD Horizon™	Cat#562916
Mouse anti-human CD45-FITC	BD Pharmingen™	Cat#555482
Mouse anti-human CD11b -PE-Cy™	BD Pharmingen™	Cat#555389
Mouse anti-human CD33-PE-Cy5™	BD Pharmingen™	Cat#551377
Mouse anti-human CD15-PE-Cy5™	BD Pharmingen™	Cat# 557744
Mouse anti-human CD66b-PE	BD Pharmingen™	Cat#561650
Mouse anti-human MPO-FITC	Invitrogen	Cat#11-1299-42
Mouse anti-human Lactoferrin-PE	Life Technologies	Cat#GIC206
Mouse anti-human CD16 FITC	BD Pharmingen™	Cat#555406

**Chemicals, Peptides, and Recombinant Proteins**		

TeSR™-E8™ Basal Medium	STEMCELL™ Technologies	Cat#05990
TeSR™-E8™ 25× supplement	STEMCELL™ Technologies	Cat#05992
0.5 M EDTA pH8.0	Fisher Bioreagents	Cat#BP2482
DPBS, without CalCl_2_, MgCl_2_	Millipore Sigma	Cat#D1408
StemSpan™ H3000 medium	STEMCELL™ Technologies	Cat#09850
GlutaMAX™- I 100×	Life Technologies	Cat#35050
EX-CYTE®	MilliporeSigma	Cat#81-129-1
Human G-CSF	Amgen	Neupogen (filgrastim), G-CSF (480 μg) syringe.
Am580 retinoic acid agonist	Cayman Chemical	Cat#15261
Gentamycin solution	MilliporeSigma	Cat#G1272
Matrigel™, Growth Factor Reduced	BD Biosciences®	Cat#354230
Collagen IV from human placenta	MilliporeSigma	Cat#C5533
Y-27632 Dihydrochloride	Peprotech	Cat#1293823
HyQTase *(discontinued)*	GE Healthcare Life Sciences	Cat#SV30030.02
Stemline® II Hematopoietic Stem Cell Expansion Medium	Sigma	Cat#S192
Human GM-CSF	Berlex Laboratories	Cat#8914704 / Leukine (Sargramostim) (GM-CSF) (250 μg vial)
UM171	Xcess Biosciences	Cat#M60223-2
Wright-Giemsa solution	MilliporeSigma	Cat#WG128
MethoCult™ H4435 Enriched	STEMCELL™ Technologies	Cat#04435
TransIT®-mRNA Kit	Mirus	Cat#MIR 2250
Ghost Dye™ Violet 540	Tonbo Biosciences	Cat#13-0879
DMSO	Sigma	Cat#D2650
Shrimp Alkaline Phosphatase (rSAP)	NEB	Cat#M0371
FGF2	Peprotech	Cat#100-18B

**Experimental Models: Cell lines**		

Bone marrow-derived IISH1i-BM1 hiPSCs and IISH2i-BM9 hiPSCs	Hu et al., 2011	WiCell (Madison, WI)
Fibroblast- derived DF19-9-7T hiPSCs	Yu et al., 2009	WiCell (Madison, WI)
Human *ETV2* transcript variant 1	*n/a*	NM_014209.3

**Recombinant DNA**		

pGEM-T Easy	Promega	Cat#A1360

**Other**		

GeneArt gene synthesis	Thermo Fisher Scientific	n/a
Phusion® HiFi DNA poly	NEB	Cat#M0530L
QIAEX II gel extraction kit	Qiagen	Cat#20021
MEGA script T7 kit	ThermoFisher	Cat#AM1334
Pseudouridine triphosphate	TriLink Biotechnologies	Cat#N-1019-5
PureLink RNA Micro kit	Thermo Fischer Scientific	Cat#12183016
TransIT-mRNA reagent	MIRUS	Cat#MIR2250
6-well plate	Fisher	Cat#12556004
Flow tube	Fisher (Corning Falcon)	Cat#352054

**Oligonucleotides**		

**Primers**	**Source**	**Sequence**
modRNA Forward Primer	Millipore Sigma	ATCGGTGCGGGCCTCTTCGCTA
modRNA Reverse Primer	Millipore Sigma	TTTTTTTTTTTTTTTTTTTTTTTTTTTTTTTTTTTT TTTTTTTTTTTTTTTTTTTTTTTTTTTTTTTTTTTT TTTTTTTTTTTTTTTTTTTTTTTTTTTTTTTTTTTT TTTTTTTTTTTTTTTTTTTTTTTTTTTTTTTT ATATGGTCGACGCAATGAAA

## Materials and Equipments

### hiPSCs (Human Induced Pluripotent Stem Cells) Culture Medium

Combine the following reagents to prepare TeSR™-E8™ complete media and then aliquot 50 mL/tube

500 mL of TeSR™-E8™ Basal medium
20 mL of TeSR™-E8™ 25× supplement
• Store at 2°C–8°C for up to 2 weeks.

### Dissociation Solution for hiPSCs

• Mix the following reagents, filter sterilize using a 0.22 μm membrane filter, and aliquot 10 mL/tube
500 μL 0.5 M EDTA pH8.0
500 mL DPBS
• Store at 2°C–8°C for up to one month.

### hiPSC Freezing Media

• Combine the following reagents to prepare hiPSC freezing media and then aliquot 50 mL/tube
450 mL of TeSR™-E8™ Basal medium
50 mL of DMSO
• Store at 2°C–8°C for up to 2 weeks.

### Hemogenic Endothelium Media

• Mix the following reagents, filter sterilize using a 0.22 μm membrane filter, and aliquot 12 mL/tube
50 mL of Stemline® II Hematopoietic Stem Cell Expansion Medium
10 μL of 100 ng/mL Human FGF-2 (Final concentration of 20 ng/mL)
[Reconstitution of Human FGF2: Centrifuge the vials at maximum speed for 1 min to precipitate lyophilized pellet prior to opening vials. Reconstitute in 5 mM Tris, pH 7.6. according to the product information provided by manufacturer. Dilute with 0.1% BSA/PBS solution for working concentration and store at −80°C until needed for use.]
• Store at 4°C for up to 2 weeks.

### Hematopoietic Differentiation Media

• Mix the following reagents, filter sterilize using a 0.22 μm membrane filter, and aliquot 12 mL/tube
50 mL of Stemline® II Hematopoietic Stem Cell Expansion Medium
10 μL of 100 ng/mL Human FGF-2 (Final concentration of 20 ng/mL)
12.5 μL of 100 ng/mL Human GM-CSF (Final concentration of 25 ng/mL)
[Reconstitution of Human GM-CSF: Centrifuge the vials at maximum speed for 1 min to precipitate lyophilized pellet prior to opening vials. Reconstitute in sterile water according to the product information provided by manufacturer. Dilute with 0.1% BSA/PBS solution for working concentration and store at −80°C until needed for use.]
0.714 μL of 3.5 mM UM171 (Final concentration of 50 nM)
[Reconstitution of small molecule UM171: Centrifuge the vials at maximum speed for 1 min to precipitate lyophilized pellet prior to opening vials. Reconstitute in DMSO according to the product information provided by manufacturer. Dilute with 0.1% BSA/PBS solution for working concentration and store at −80°C until needed for use.]
• Store at 4°C for up to 2 weeks.

***Note:*** UM171 does not increase the number of myeloid cells, but significantly improves neutrophil generation.

### Neutrophil Differentiation Media

• Combine the following reagents, filter sterilize using a 0.22 μm membrane filter, and aliquot 10 mL/tube
• 494 mL StemSpan™ H3000 medium
• 5 mL GlutaMAX™-I 100× (Final concentration 1×)
• 1 mL 100% EX-CYTE (Final concentration 0.2%)
• 750 μL 100 ng/mL Human G-CSF (Final concentration 150 ng/mL)
[Reconstitution of Human G-CSF: Centrifuge the vials at maximum speed for 1 min to precipitate lyophilized pellet prior to opening vials. Reconstitute in sterile water according to the product information provided by manufacturer. Dilute with 0.1% BSA/PBS solution for working concentration and store at −80°C until needed for use.]
• 125 μL 10 mM Am580 retinoic acid agonist (Final concentration 2.5 μM)
[Reconstitution of small molecule Am580: Centrifuge the vials at maximum speed for 1 min to precipitate lyophilized pellet prior to opening vials. Reconstitute in DMSO according to the product information provided by manufacturer. Dilute with 0.1% BSA/PBS solution for working concentration and store at −80°C until needed for use.]
Gentamycin solution
• Store at 2°C–8°C for up to 2 weeks.

### Flow Cytometry Buffer

• Combine the following reagents, filter sterilize using a 0.22 μm membrane filter, and aliquot 10 mL/tube
○ 488 mL 1× DPBS
○ 10 mL FBS
○ 0.25 g Sodium azide (NaN_3_)
○ 2 mL 0.5 M EDTA (pH 8.0)
• Store at 4°C up to 6 months.

### Selection of hiPSC Lines

Bone marrow-derived IISH2i-BM9 and IISH1i-BM1 hiPSCs (Hu et al., 2011)

Fibroblast- derived DF19-9-7T hiPSCs (Yu et al., 2009).

These cell lines are provided by WiCell (Madison, WI).

## Step-By-Step Method Details

### Maintenance of hiPSC

**Timing: ∼25 min**

**Figure 2:**
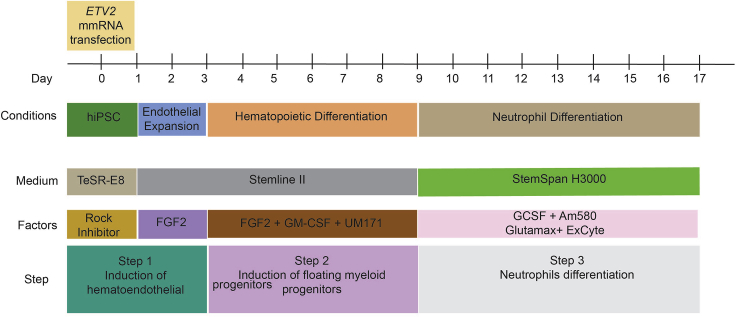
Schematic Diagram of Protocol for Generation of Neutrophils from hiPSCS in Defined Serum Free and Feeder-Free Conditions

hiPSCs are maintained under feeder-free conditions on Matrigel in TeSR™-E8™ complete media (Figure 2).

1. Retrieve a Matrigel solution containing 6-well plate from 4°C storage and incubate at 37°C for minimum 30 min to 1–2 h before thawing the iPSCs.

***Note:*** Store Matrigel solution containing plates in 4°C refrigerator for overnight. These plates can be stored in refrigerator for 1 month.

2. Aspirate the Matrigel from each well and put 1 mL of prewarmed TeSR™-E8™ complete media in each well.
3. Add 5 mL of TeSR™-E8™ complete media to a 15 mL conical tube.
4. Thaw cryovial of hiPSC from liquid nitrogen storage in a 37°C water bath.

**CRITICAL:** Proceed to step 5 immediately after complete thaw. Avoid keeping cryovial in water bath for extended period time, which will compromise cell viability.

5. Add 1 mL of TeSR™-E8™ complete media into the cryovial, gently mix and collect the total 6 ml cell suspension in the 15 mL tube from step 3.

**CRITICAL:** Mix cells very gently. Avoid vigorous mixing.

6. Centrifuge at 150×*g* for 5 min at room temperature and aspirate the supernatant.
7. Resuspend the cell pellet with TeSR™-E8™ complete media with 10 μM Rock inhibitor Y-27632 Dihydrochloride.
8. Gently rock the plate back-and-forth to coat the cells evenly over the wells, and place in 37°C, 5% CO_2_, incubator.
9. Change the media with fresh TeSR™-E8™ complete media one day after seeding to remove Rock inhibitor.
10. Replenish the media every 48 h with TeSR™-E8™ complete media.

### Passage of hiPSCs

**Timing: ∼30 min**

hiPSCs are passaged when cells achieve 60%–80% confluency (typically every 5–6 days).

**Figure 3:**
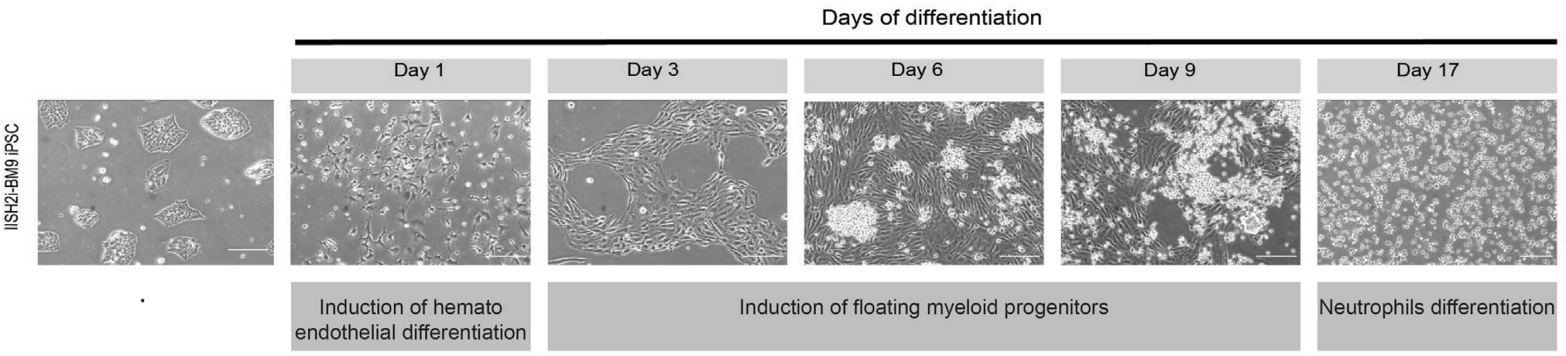
Representative Phase Contrast Images Showing Difference in Morphology during the Hematoendothelial Development and Neutrophil Differentiation following Transduction of IISH2i-BM9 hiPSCs with *ETV2* mmRNA

11. Retrieve a Matrigel-coated 6-well plate from 4°C storage and incubate at 37°C, 5% CO_2_, for at least 30 min before passaging iPSCs.
12. Aspirate the Matrigel and put 1 mL prewarmed TeSR™-E8™ complete media into each well.
13. When iPSCs are 60%–80% in confluency (Figure 3), aspirate the media and add 1 mL prewarmed dissociation solution (0.5 mM EDTA/1× PBS).

**CRITICAL:** The cells should be passaged every 5–6 days . Do not allow hiPSC cultures to become more than 80% confluent.

14. Incubate at 37°C, 5% CO_2_, for 3 min and slowly aspirate the dissociation solution.
15. Add 2 mL of prewarmed TeSR™-E8™ complete media into each well of a 6-well plate and break the colonies into small aggregates by gentle pipetting.

**CRITICAL:** Do not use excessive mechanic force while pipetting.

16. Collect the cells into a 15 mL conical tube and centrifuge at 350×*g* for 5 min at room temperature and aspirate the supernatant.

***Note:*** Centrifugation speed is higher than that of the speed during thawing of hiPSCs. During thawing procedure cells are in very labile condition in DMSO containing freezing media. So, centrifugation speed is low for not to harm the cells.

17. Resuspend the cells in 6 mL of TeSR™-E8™ complete media.
18. Plate 1 mL of cell suspension in each well of a 6-well plate containing 1 mL of TeSR™ -E8^TM.^ complete media.

***Note:*** Split the cells from one well of 6-well plate into 6 wells of a 6-well plate with TeSR™ -E8™ complete media without Rock inhibitor.

19. Gently rock the plate side-to-side to coat the cells evenly over the wells and place in 37°C, 5% CO_2_, incubator.
20. Replenish the media every 48 h with TeSR™-E8™ complete media.

***Note:*** The undifferentiated hiPSC colonies must be composed of tightly packed cells with prominent nucleoli. They should have well-defined sharp edges (Figure 3). Then they are ready to use for differentiation. Passage no lower than 30 is recommended to use for differentiation. If cells are not having well-defined sharp edges, then these cells are considered as bad hiPSCs. These cells do not show good mmRNA transfection efficiency.

### Cryopreservation of hiPSCs

**Timing: ∼30 min**

Cryopreservation of hiPSCs is performed in hiPSC freezing media to maintain the batch of hiPSCs for future differentiation.

21. When hiPSCs cell density reaches 60%–80%, aspirate the media and add 1 mL prewarmed dissociation solution (0.5 mM EDTA/1× PBS).
22. Incubate at 37°C, 5% CO_2_, for 3 min and slowly aspirate the dissociation solution.
23. Add 2 mL of prewarmed TeSR™-E8™ complete media into each well of a 6-well plate and break the colonies into small aggregates by gentle pipetting.
24. Collect the cells into a 15 mL conical tube and centrifuge at 350×*g* for 5 min at room temperature and aspirate the supernatant.
25. Resuspend the cells in 6 mL of hiPSC freezing media and distribute 1 ml of cell solution in each cryovials.

***Note:*** Each cryovial must contain minimum 1×10^6^ cells.

26. Store cryovials are in isopropanol containing coolant in −80°C for overnight.

***Note:*** Do not keep cryovials at −80°C not more than a week.

27. Transfer the cryovials into liquid nitrogen tank for long term storage.

### Transfection of *ETV2* Modified mRNA (mmRNA) into hiPSCs and Induction of Hemogenic Endothelial Lineage

**Timing: ∼2 h**

To induce hemogenic endothelium, hiPSCs are transfected with *ETV2* mmRNA in TeSR™-E8™ complete media using TransIT reagent and mRNA boost.

**Figure 4:**
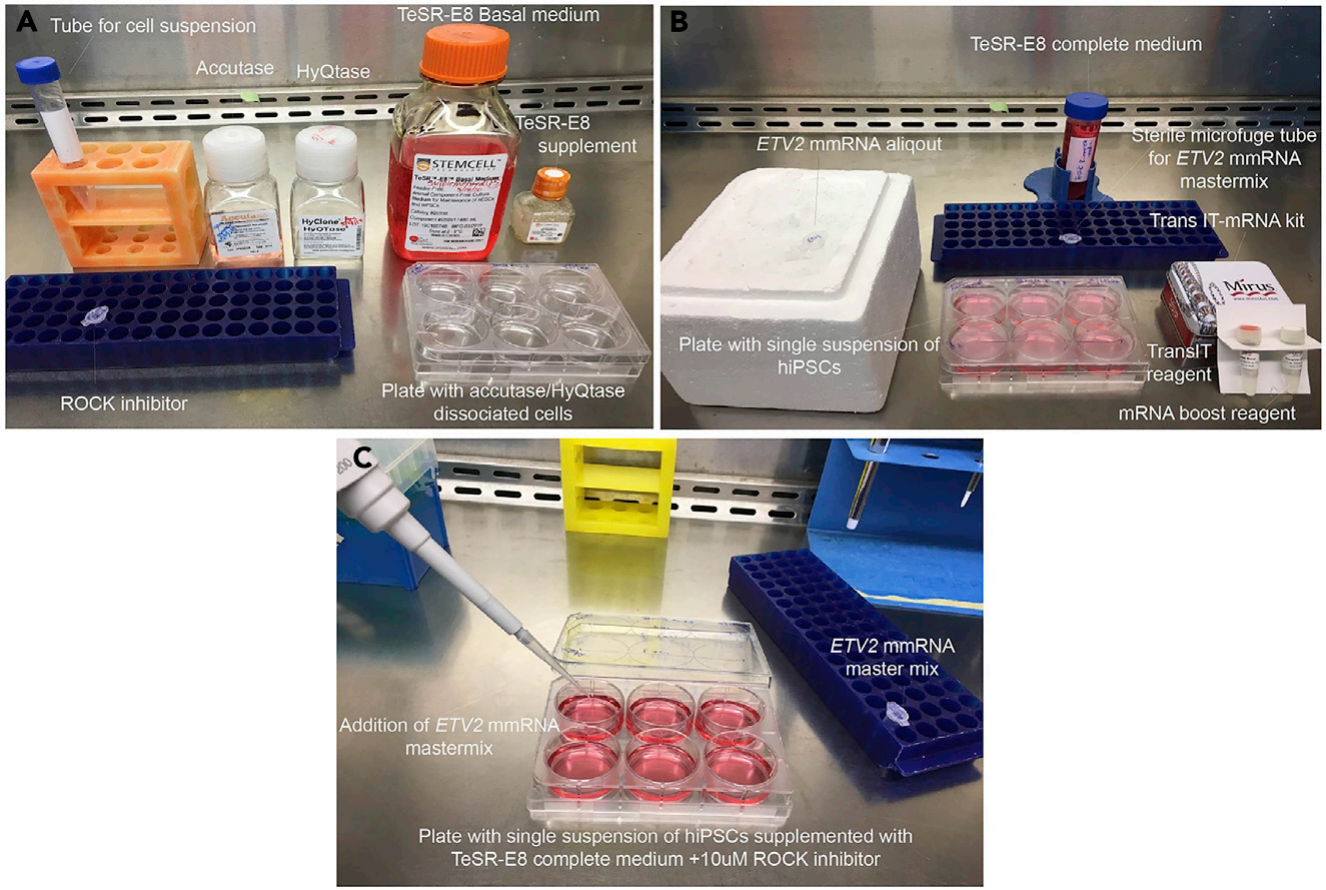
Preparation of Cell Culture Hood Set up for Single Cell Suspension and Transfection Procedure (A) Suggested cell culture hood set up for the single cell suspension of hiPSCs. (B) Recommended cell culture hood set up for the transfection of the *ETV2* mmRNA transfection. (C) Addition of the *ETV2* mmRNA master mix in to the respective wells of hiPSCs.

28. On day 0, retrieve collagen IV-coated 6-well plate from 4°C storage and keep at 37°C for at least 30 min before starting the differentiation of iPSCs.
29. Aspirate the collagen and put 2 mL prewarmed TeSR™-E8™ complete media supplemented with 10 μM Y-27632 Dihydrochloride into each well.
30. When iPSCs are 60% in confluency after 3 days of culture (Figure 3), aspirate the media and add 1 mL prewarmed HyQTase for single cell suspension of the iPSCs (Figure 4A).

***Note:*** Single cell suspension requires HyQTase instead of EDTA/PBS solution. EDTA/PBS does not make cells into single cell suspension. It only maintains the cells as clumps which is required for passaging of hiPSCs. But for differentiation, it needs single cell suspension.

31. Incubate at 37°C, 5% CO_2_, incubator for 3 min.

**CRITICAL:** Incubation should not exceed more than 3 min and should be optimized for each type of cell line. After 3 min of incubation you can observe single cells in the plate under the microscope. HyClone™ HyQTase™ solution has since been discontinued by GE Healthcare Life Sciences, therefore, we recommend to use Accutase® (Innovative Cell Technologies, Inc., Cat. #AT-104) instead.

32. Add 1 mL of prewarmed TeSR™-E8™ complete media into each well.
33. Collect the cells into a 15 mL conical tube and centrifuge at 350×*g* for 5 min at room temperature and aspirate the supernatant.

**CRITICAL:** Do not aspirate the HyClone™ HyQTase™ solution from the plate.

***Optional:*** HyClone™ HyQTase™ has since been discontinued by GE Healthcare Life Sciences. We therefore recommend to use Accutase® (Innovative Cell Technologies, Inc., Cat. #AT-104) instead.

34. To remove residual HyQTase™, resuspend the cells in 2 mL of TeSR™-E8™ complete media.
35. Centrifuge at 350×*g* for 5 min at room temperature and aspirate the supernatant.
36. Resuspend the cells in 1 mL of TeSR™-E8™ complete media.
37. Determine cell viability by trypan blue staining and a hemocytometer.
38. Plate 2×10^5^ cells into each well of a 6-well plate containing 2 mL of TeSR™-E8™ complete media with 10 μM Rock inhibitor and gently rock the plate side-to-side and back-and-forth to spread the cells evenly across the well.

**CRITICAL:** Cell number should be highly accurate as transfection efficiency is highly dependent on cell density and viability.

39. Incubate the plate at 37°C, 5% CO_2_, for 30 min to adhere the cells.
40. Place the TransIT reagent and mRNA boost (TransIT®-mRNA Kit, Mirus) and *ETV2* mmRNA aliquot on ice (Figure 4B).
41. Prepare *ETV2* mmRNA transfection master mix in a sterile 1.5 mL microcentrifuge tube.
42. Mix the components by gentle pipetting and add the ∼100 μL transfection mixture to each well of the 6-well plate (Figure 4C).

**CRITICAL:** Add the transfection master mix drop by drop into each well evenly. Rock the plate after addition to properly distribute.

43. Place in 37°C, 5% CO_2_, incubator for 24 h.

**CRITICAL:** We recommend not to use an iPSC cell line beyond passage 30, after which, hematopoietic differentiation capacity reduces.

### Expansion of Hemogenic Endothelial Cells

**Timing: ∼2 days**

After *ETV2* mmRNA transfection, cells are cultured in hemogenic endothelium medium (StemLine II with FGF2) to support formation and expansion of hemogenic endothelial cells.

44. On day 1, after 24 h of transfection, observe cell morphology under phase contrast microscopy (Figure 3) and then aspirate the media and add 2 mL of prewarmed hemogenic endothelium media.

***Note:*** Transfected cells shows a star shaped morphology- thin protrusions on the periphery. Cells other than this morphology are not transfected cells.

45. Place in 37°C, 5% CO_2_, for 24 h.
46. On day 2, aspirate media and add 2 mL of prewarmed hemogenic endothelium medium again.

***Optional:*** We recommend to use enhanced green fluorescence protein (eGFP) mmRNA as a positive control to assess the transfection efficiency.

***Optional:*** We also advise to check the endothelial marker CD144 expression on day 1. *ETV2* mmRNA transfected cells show more than 90% CD144 expression.

### Generation of Myeloid Progenitors

**Timing: ∼7 days**

Culture of *ETV2*-induced hemogenic endothelium in hematopoietic differentiation medium (StemLineII with FGF2, GM-CSF, and UM171) promotes endothelial-to-hematopoietic transition and formation of CD34^+^CD33^+^ myeloid progenitor cells enriched in granulocytic progenitors.

47. On day 3, prepare hematopoietic differentiation media and observe cell morphology under phase contrast microscopy (Figure 3).
48. Aspirate the supernatant and add 2 mL of prewarmed hematopoietic differentiation media. Place at 37°C, 5% CO_2_.
49. Add 1 mL hematopoietic differentiation media on the top of the existing media without removing the media every 48 h up to day 8.
50. On day 9, observe the morphology of the cells under phase contrast microscopy (Figure 3).
51. Collect floating myeloid progenitors on day 9 into a 15 ml conical tube and transfer to neutrophil differentiation conditions.

***Note:*** After the first collection of floating cells on day 9, adherent cells continue to produce myeloid progenitors. Adding of 2 mL hematopoietic differentiation media every 2 days to the remaining adherent cells allows for additional two rounds of myeloid progenitor collection between days 16 and 23.

***Note:*** Adherent cells are hemogenic endothelial cells, which give rise to floating myeloid progenitors. UM171 is one of the important small molecules in hematopoietic differentiation media. It does not increase the number myeloid cells. However, it does significantly improve neutrophil output.

### Neutrophil Differentiation

**Timing: ∼8 days**

Myeloid progenitors are differentiated into fully functional neutrophils in a neutrophil differentiation medium (StemSpan H3000 with G-CSF and retinoic acid receptor α agonist Am580).

52. Centrifuge collected floating myeloid progenitor cells at 350×*g* for 5 min at room temperature and aspirate the supernatant.
53. Resuspend the cells in 2 mL of neutrophil differentiation media to remove any residual hematopoietic differentiation media.
54. Centrifuge at 350×*g* for 5 min at room temperature and aspirate the supernatant.
55. Resuspend the cells in 1 mL of neutrophil differentiation media.
56. Determine the cell viability by using trypan blue and a hemocytometer.
57. To induce neutrophil differentiation, plate 5×10^5^ cells in one well of a 6-well plate containing 4 mL neutrophil differentiation media and gently rock the plate side-to-side, back-and-forth to spread the cells evenly across the wells.
58. After 4 days, add fresh 2 mL of fresh neutrophil differentiation media on the top of the existing culture.
59. After 8 days of differentiation in neutrophil differentiation media observe the morphology of the cells under phase contrast microscopy on day 17 (Figure 3).
60. Harvest the neutrophils from the supernatant, leaving adherent progenitors and macrophages.

***Note:*** Neutrophils have to be generated and used for functional analysis fresh. Although neutrophils do not survive freezing in standard freezing media, myeloid progenitors generated in step 51 can be cryopreserved in standard conditions and used for neutrophil generation after thawing.

### Colony-Forming Cell Assay of (CFC) Myeloid Progenitors

**Timing: ∼13 days**

Colony-Forming Cell (CFC) assay is used to determine formation of myeloid progenitors in cultures on day 9 of differentiation.

61. Thaw 3 ml aliquots of CFC assay media (MethoCult™ H4435 Enriched).
62. Suspend 3×10^3^ floating myeloid progenitor cells to a 3 ml aliquot of Methocult™ H4435.
63. Vortex vigorously. Let the tube stand still for 15 min at 37°C water bath.
64. Attach a 16-gauge blunt-end needle to a 3 ml syringe and draw up 2.2 ml. Do not draw up large bubbles; expel them at the beginning by pushing out a couple of times. Push out 1.1 ml each into two 30 mm non-treated dish and spread out the mixture evenly by rotating.
65. Place duplicate plates in a 100 mm plate together with a water dish containing 3 ml sterile water. Culture for 13 days.
66. Characterize and score the colonies according to their morphology with a bright field microscope in a culture dish marked with a scoring grid.

### Flow Cytometric Analysis of Myeloid Progenitors and Neutrophils

**Timing: ∼60 min**

Flow cytometric analysis is used to identify the population of interest among a mixture of different populations.

67. Cell suspension to a concentration of 1×10^6^ cells/mL in flow cytometry buffer. Cells are usually stained in polystyrene round bottom falcon tubes.
68. Add 0.1–10 μg/mL conjugated primary antibody.

***Note:*** Dilutions should be made in flow cytometry buffer (Ghost dye™ Violet 540 can also be added at this point for dead cell exclusion).

69. Incubate for at least 30 min in dark at 4°C.
70. Wash the cells by centrifugation at 350×*g* for 5 min and resuspend them in 500 μL of flow cytometry buffer.
71. Analyze the cells on the flow cytometer.

## Expected Outcomes

*ETV2* mmRNA induction produces 1.7×10^7^ neutrophils from 10^6^ hiPSCs within 3 weeks.

These neutrophils can be tested in the following ways:

### Morphology

Undifferentiated hiPSC colonies composed of tightly packed cells with prominent nucleoli and have well-defined sharp edges (Figure 3). After transfection of singularized iPSCs with *ETV2* mmRNA and culture in endothelial expansion medium, cells acquire a typical endothelial morphology within 72 h (Figure 3). During the process of differentiation, hemogenic endothelial cells undergo endothelial-to-hematopoietic transition and form floating myeloid progenitors (Figure 3). Neutrophil differentiation can be confirmed by morphologic analysis of Wright-Giemsa stained cytospins prepared from floating cells. Neutrophils have a characteristic multilobed nucleus with cytoplasm containing purplish granules (Figure 6A).

### Hematopoietic Colony-Forming Potential

**Figure 5:**
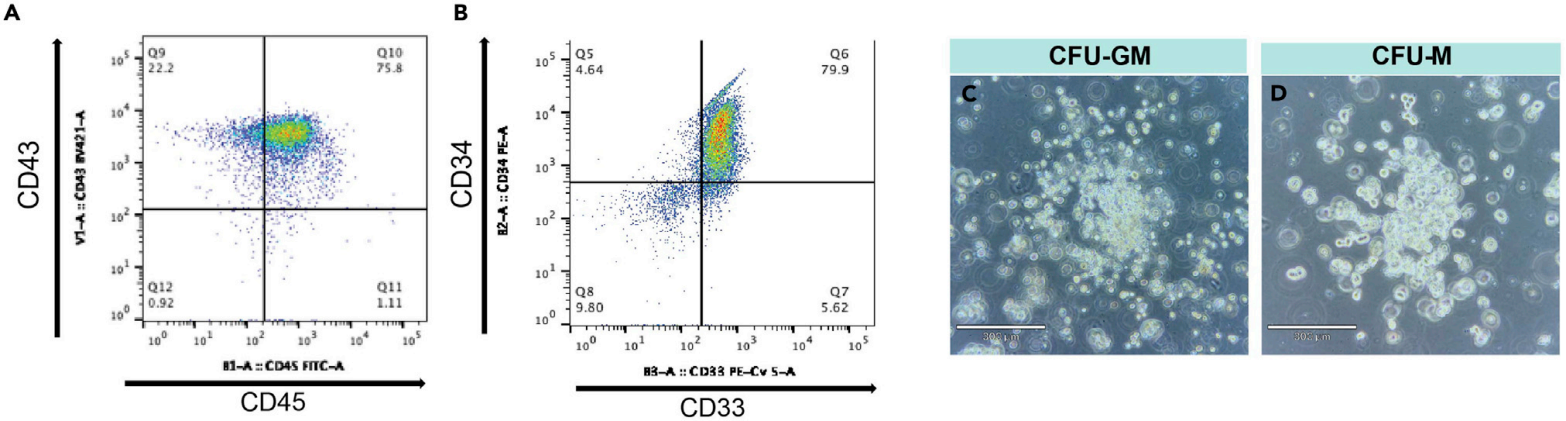
Formation of Myeloid Progenitors in hiPSCs Transfected with *ETV2* mmRNA (A) Flow cytometric analysis of CD43, and CD45 expression in myeloid progenitors generated from *ETV2* mmRNA transfected IISH2i-BM9 hiPSCs on day 9. (B) Flow cytometric analysis of CD33 and CD34 expression in myeloid progenitors. (C) Representative image of CFU-GM colony in CFC assay of myeloid progenitors formed in culture. (D) Representative image of CFU-M colony in CFC-assay of myeloid progenitors.

The differentiation ability of myeloid progenitors can be determined by Colony-Forming Cell (CFC) assay. In this protocol myeloid progenitors produce CFU-Macrophage (CFU-M) (Figure 5D) and CFU- Granulocyte Macrophage (CFU-GM) colonies (Figure 5C) in CFC analysis. [https://doi.org/10.1016/j.stemcr.2019.10.007]

### Cell Surface Markers

**Figure 6:**
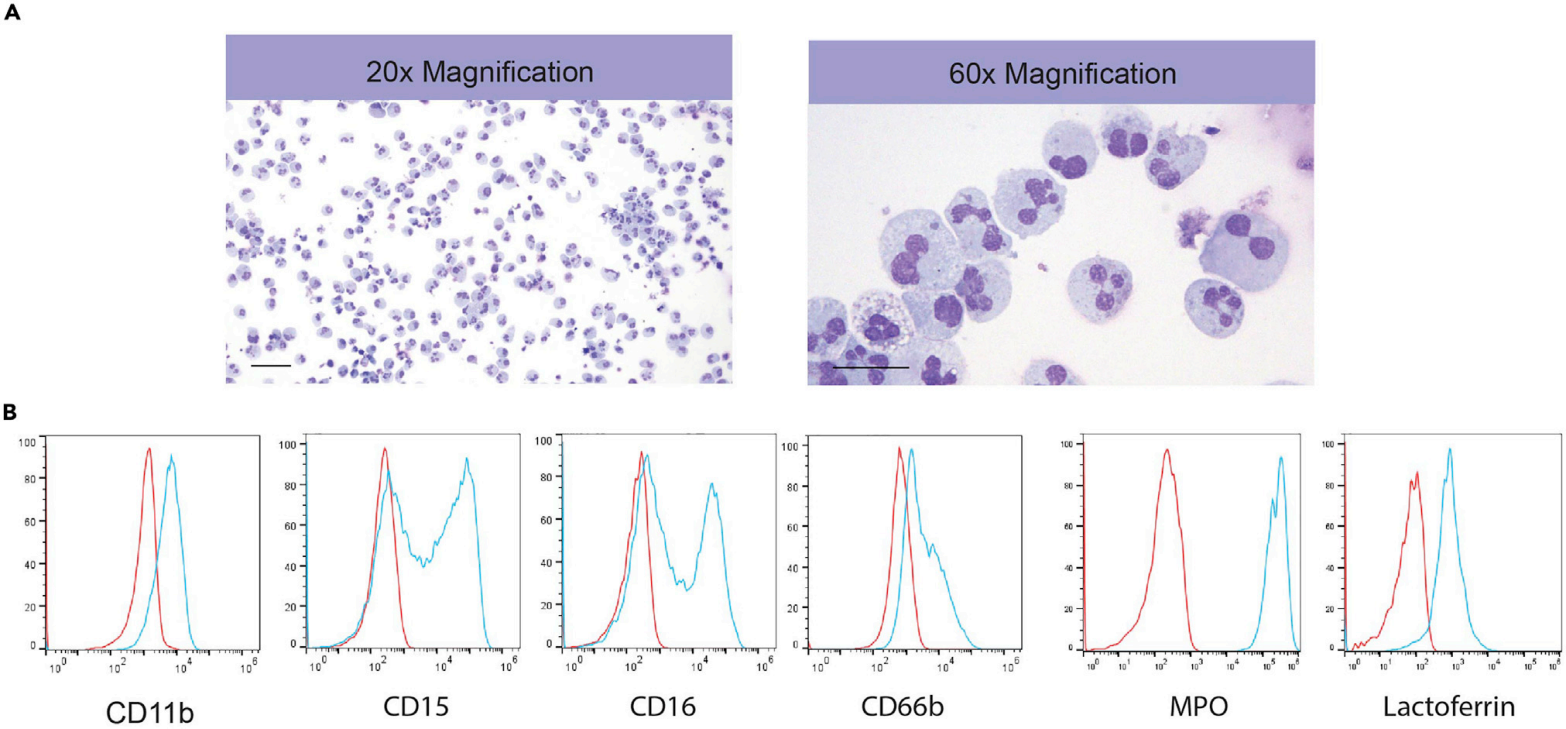
Induction of Neutrophil Formation from Myeloid Progenitors (A) Representative images of Wright stained cytospins showing the morphology of neutrophils. (B) Flow cytometric analysis of CD11b, CD15, CD16, CD66b, MPO and lactoferrin expression in generated neutrophils.

Myeloid progenitors express CD33, CD34, CD43, CD45 surface markers on day 9 (Figures 5A and 5B). Surface expression of CD11b, CD16, CD15, CD66b, and intracellular expression of MPO and lactoferrin (Figure 6B), in terminally differentiated cells proves the successful generation of neutrophils.

[https://doi.org/10.1016/j.stemcr.2019.10.007]

### Other Evaluations

Functionality of generated neutrophils can be assessed by analyzing phagocytosis, reactive oxygen species (ROS) generation, migration and neutrophil extracellular trap (NET) formation assay.

## Limitations

Our protocol produces a relatively homogenous population of neutrophils from different hESC and iPSC lines. However, differentiation efficacy can vary between lines. Optimization of *ETV2* mmRNA transfection, cell densities and time frames at which differentiation steps are initiated, may all be required when other PSC lines are used. Using iPSCs beyond passage 30 is not recommended as the differentiation capability is reduced in feeder-free conditions. Transfection of mmRNA requires adequate skills and experience. Thus, preliminary evaluation of mmRNA transfection efficacy using *eGFP* mmRNA is recommended. Neutrophils generated by this protocol phagocytose bacteria and efficiently produce ROS. However, they are closer to fetal than adult neutrophils, and their NET production in response to PMA and chemotactic response to IL-8 is somewhat impaired in comparison with adult peripheral blood neutrophils.

## Troubleshooting

### Problem 1

mmRNA Transfection Procedure.

[Link: Step-By-Step Method Details steps 40–42]

### Potential Solution 1

My mmRNA transfection failed sometimes due to higher than passage no 30. So, it is important to use low passage iPSCs for differentiation in this feeder-free condition. Also, my experiment failed sometimes due to incubation of the mmRNA transfection master mix more than 3 min at room temperature. Therefore, the incubation of mmRNA with transfection master mix at room temperature should not exceed more than 3 min. On the other hand, one of the reasons behind the failure, could be the addition of the mmRNA transfection master mix at the center of the plate without mixing. It is critical to distribute master mix into plate equally over the plate by adding drop by drop while rocking the plate side-by-side and back-and-forth.

### Problem 2

Cell Density.

### Potential Solution 2

It is crucial to maintain proper cell density. Cells should be plated as a single cell suspension and cell density must be maintained at 2×10^5^ cells in each well of a 6-well plate. More than 2×10^5^ cells can hamper transfection efficiency of mmRNA.

### Problem 3

mmRNA stability.

### Potential Solution 3

It is important to make aliquots of mmRNA and store them at −80°C. It is necessary to take out the aliquots on ice to maintain mmRNA integrity. Avoid freeze-thaw by making aliquot volumes small enough for single use.

### Problem 4

Transfection efficiency.

### Potential Solution 4

It is recommended to use *eGFP* mmRNA as a positive control to assess the transfection efficiency with mmRNA. More than 90% of GFP-positive cells should be observed in iPSC cultures 24–48 h after transfection with eGFP mmRNA.

### Problem 5

ETV2 expression.

### Potential Solution 5

It is advisable to analyze the ETV2 protein expression level after 24 h of *ETV2* mmRNA transfection by Western Blot.

### Problem 6

Cytokine stability.

### Potential Solution 6

Cytokines must be stored at −80°C for long term storage. It is necessary to take out the aliquots on ice. Avoid freeze-thaw by making aliquot volumes small enough for single use.

## Resource Availability

### Lead Contact

Further information and requests for resources should be directed to the Lead Contact Igor Slukvin (islukvin@wisc.edu).

### Materials Availability

This protocol did not generate any new data or code.

### Data and Code Availability

This protocol did not generate any new data or code.
